# *In Vitro* Expanded Stem Cells from the Developing Retina Fail to Generate Photoreceptors but Differentiate into Myelinating Oligodendrocytes

**DOI:** 10.1371/journal.pone.0041798

**Published:** 2012-07-25

**Authors:** Magdalena Czekaj, Jochen Haas, Marlen Gebhardt, Thomas Müller-Reichert, Peter Humphries, Jane Farrar, Udo Bartsch, Marius Ader

**Affiliations:** 1 Center for Regenerative Therapies Dresden (CRTD), TU Dresden, Dresden, Germany; 2 Eye Clinic, University Medical Centre Hamburg-Eppendorf, Hamburg, Germany; 3 Smurfit Institute of Genetics, Trinity College Dublin, Dublin, Ireland; 4 Medical Theoretical Center, TU Dresden, Dresden, Germany; Seattle Children’s Research Institute, United States of America

## Abstract

Cell transplantation to treat retinal degenerative diseases represents an option for the replacement of lost photoreceptor cells. *In vitro* expandable cells isolated from the developing mammalian retina have been suggested as a potential source for the generation of high numbers of donor photoreceptors. In this study we used standardized culture conditions based on the presence of the mitogens FGF-2 and EGF to generate high numbers of cells *in vitro* from the developing mouse retina. These presumptive ‘retinal stem cells’ (‘RSCs’) can be propagated as monolayer cultures over multiple passages, express markers of undifferentiated neural cells, and generate neuronal and glial cell types upon withdrawal of mitogens *in vitro* or following transplantation into the adult mouse retina. The proportion of neuronal differentiation can be significantly increased by stepwise removal of mitogens and inhibition of the notch signaling pathway. However, ‘RSCs’, by contrast to their primary counterparts *in vivo*, i.e. retinal progenitor cells, loose the expression of retina-specific progenitor markers like *Rax* and *Chx10* after passaging and fail to differentiate into photoreceptors both *in vitro* or after intraretinal transplantation. Notably, ‘RSCs’ can be induced to differentiate into myelinating oligodendrocytes, a cell type not generated by primary retinal progenitor cells. Based on these findings we conclude that ‘RSCs’ expanded in high concentrations of FGF-2 and EGF loose their retinal identity and acquire features of *in vitro* expandable neural stem-like cells making them an inappropriate cell source for strategies aimed at replacing photoreceptor cells in the degenerated retina.

## Introduction

Visual impairment and blindness due to photoreceptor loss in degenerative retinal disorders, such as age-related macula degeneration (AMD) or retinitis pigmentosa (RP), is one of the prime reasons for disability in industrialized countries. Diverse therapeutic strategies [1] are under investigation including pharmacological procedures [2], [3], prosthesis implantations [4], or gene therapeutic strategies [5]–[7]. Another approach represents the transplantation of cells to replace lost photoreceptors. Indeed, proof of concept studies in mice demonstrated that photoreceptor precursors can integrate into the adult mammalian retina and form mature and functional photoreceptors [8]–[12]. However, the optimal developmental time point for isolation of transplantable photoreceptor precursors in the mouse is around postnatal day 4 [8], [9]. It corresponds to the second trimester in humans, therefore the use of such primary cells from humans for therapies is substantially limited due to logistic difficulties beside possible ethical concerns and problems associated with supply of sufficient numbers of donor cells. Thus, the expansion of cells *in vitro* that have the capacity to generate mature photoreceptors after transplantation and therefore provide an unlimited amount of donor material represents a vital step for future therapeutic applications.

During the last two decades methods for the *in vitro* expansion of stem/progenitor cells (called neural stem cells; NSCs) from the central nervous system (CNS) have been established [13]–[15]. However, culturing conditions of NSCs based on high, unphysiological concentrations of the mitogens FGF-2 and EGF results in loss of regional identity over time/passages [16]–[18] suggesting that *in vitro* propagated NSCs are distinct from endogenous stem/progenitor populations [19]. In the retina field, several studies suggested that presumptive ‘retinal stem cells’ (‘RSCs’), isolated from the developing neuroretina or pigmented ciliary margin of mammals and expanded under similar culture conditions as NSCs, are multipotent and have the potential to differentiate into all principle retinal cell types, including photoreceptors [20]–[24]. However, photoreceptor generation was mainly demonstrated by immunostainings or RT-PCR. Importantly, morphological evidence for differentiation into mature photoreceptors containing outer segments and axonal terminals, as it was shown for transplanted primary photoreceptor precursors [8], [9], [11], [12], has not been reported following ‘RSCs’ transplantation [20], [21], even when ‘RSCs’ were pre-differentiated *in vitro* [23]. Two recent studies reported that cells generated from the adult mammalian pigmented ciliary margin and expanded as ‘RSCs’ are incapable to differentiate into photoreceptors [25], [26].

Here we show that self-renewing stem cells, isolated from the developing mouse retina and expanded *in vitro* as monolayers in the presence of EGF and FGF2, loose retinal features as they down-regulated the expression of genes characteristic for retinal progenitor cells and failed to differentiate into photoreceptors *in vitro* or following transplantation *in vivo*. Moreover, we provide novel evidence that expanded retinal cells acquire a differentiation potential similar to NSCs with the ability to generate not only astrocytes and neurons, but also myelinating oligodendrocytes - a cell type that is normally neither generated nor present in the mouse retina [27]–[30].

## Results

### Cells Isolated from the Developing Retina are Highly Expandable *in vitro* but Loose Expression of Retina-specific Genes

**Figure 1:**
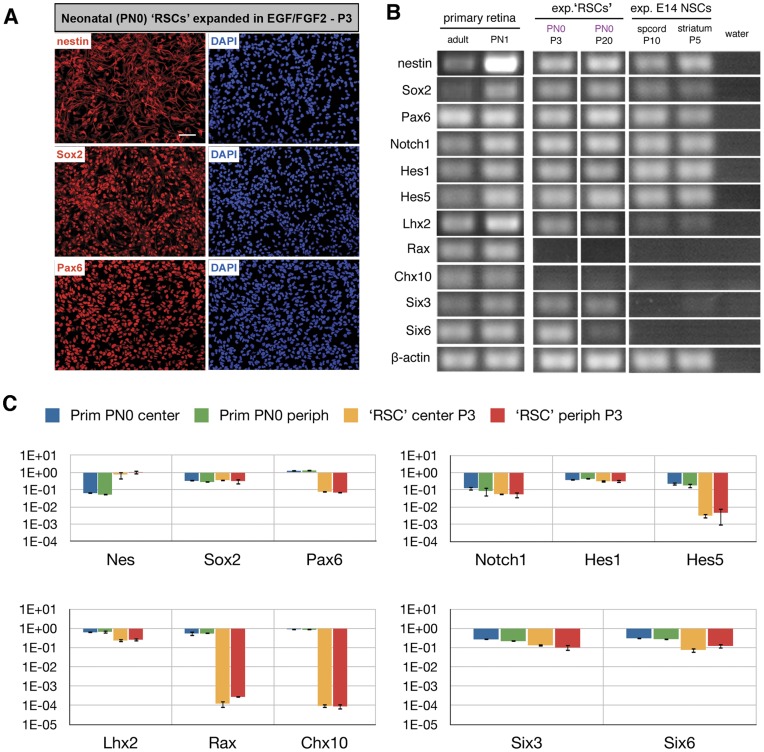
Immunocytochemistry, RT-PCR and Q-PCR analyses of neonatal ‘RSCs’ during *in vitro* cultivation. Following expansion in the presence of growth factors ‘RSCs’ show immunoreactivity for *nestin*, *Sox2* and *Pax6* at the protein (A) and mRNA levels (B, C). Further gene expression examination using semi-quantitative RT-PCR revealed that throughout all passages analyzed ‘RSCs’ express components of the notch signaling pathway (*Notch1* receptor, *Hes1* and *Hes5*). Levels of transcription factors crucial for eye and retina development (primary retina, PN1) were highly variable: *Six3* expression was stable up to P20, *Lhx2* and *Six6* levels decreased with increasing passage number, and *Rax* and *Chx10* were no longer detectable beginning with passage 3 (B). In comparison, NSCs isolated from E14.5 spinal cord or striatum and cultured for 10 or 5 passages, respectively, showed expression of *nestin*, *Sox2*, *Pax6*, and notch pathway components, but were negative for *Rax*, *Chx10*, *Six3*, and *Six6*. Adult and PN1 primary retina served as a positive or negative control (B). Q-PCR analysis performed on peripheral ‘RSCs’ from P3 and on their primary counterparts (peripheral retinal cells from PN0) confirmed the above RT-PCR results: expanded cells from low passages expressed *Nes*, *Sox2*, *Pax6*, *Notch1*, *Hes1*, *Hes5*, *Six3* and *Six6* (C). Although *Lhx2* level in P3 ‘RSCs’ was as high as in primary retinal cells, *Rax* and *Chx10* genes were undetectable (C). Gene expression levels are related to the mean expression levels of housekeeping genes. Scale bar: 50 µm. Abbreviations: E, embryonic day; exp, expanded; NSC, neural stem cells; P, passage; PN, postnatal day; ‘RSCs’, retinal stem cells; spcord, spinal cord.

Following up on recently published studies [21], [22] cells isolated from the neonatal retina were subjected to expansion conditions in the presence of the mitogens FGF-2 and EGF. Postnatal day (PN) 0–1 retinas were initially chosen as at this developmental time point the majority of primary retinal progenitor cells will differentiate along the rod photoreceptor lineage *in vivo*. The resulting expanded cells were termed ‘retinal stem cells’ (‘RSCs’) as previously suggested [22]. As revealed by immunocytochemistry the majority of mitogen expanded ‘RSCs’ expressed markers typical for stem cells of the CNS including the intermediate filament protein nestin and the transcription factors *Sox2* and *Pax6* (Figure 1A). The strong expression of these ‘stemness’ genes was further confirmed by RT-PCR (Figure 1B) and more sensitive Q-PCR, which revealed *nestin* expression in cultured cells of passage 3 (P3) around 10 fold higher and *Pax6* 10 fold lower than in primary cells from which ‘RSCs’ were developed (Figure 1C). Moreover, throughout all passages analyzed expanded ‘RSCs’ expressed components of the Notch pathway: receptor *Notch1* and its downstream targets *Hes1* and *Hes5* (Figure 1B). This gene expression pattern was similar to that of *in vitro* expanded NSCs isolated from the embryonic spinal cord or striatum (Figure 1B) in accordance with previously published studies [15], [31].

Next we analyzed the expression of transcription factors that play important roles during eye and retina development. In passaged spinal cord or striatal NSC cultures the expression of retina-associated transcription factors *Lhx2*, *Rax*, *Chx10*, *Six3* and *Six6* expression was almost undetectable or absent (Figure 1B). Importantly, *Rax* and *Chx10* genes, transcription factors expressed exclusively in most retinal progenitor cells *in vivo*, were undetectable in all analyzed ‘RSCs’ (Figure 1B, C). RT-PCR analysis demonstrated stable expression of *Six3* up to passage 20, and a decrease of *Lhx2* and *Six6* expression levels (Figure 1B). The combined data indicate that passaged ‘RSCs’ adopt an expression profile similar to NSCs to variable extent and loose the expression of genes that are characteristic for retinal progenitor cells.

Q-PCR experiments performed on higher passaged (P10) ‘RSCs’ revealed variable expression levels of *Hes5*, *Lhx2*, *Six3* and *Six6* between individual ‘RSCs’, even among cultures derived from the same source. Central retina-derived ‘RSCs’ showed variances in expression of *Lhx2* and *Six3* (12- and 10-fold, respectively), while peripheral ‘RSCs’ exhibited pronounced variability of *Six6* expression reaching 96-fold difference between distinct cultures (data not shown). Notably, cells of low passage 3 showed stable expression profiles implicating that the differences in gene expression levels between separate ‘RSCs’ of the same source were acquired in the course of *in vitro* expansion.

### ‘RSCs’ Differentiate into Glial and Neuronal Cell-types

**Figure 2:**
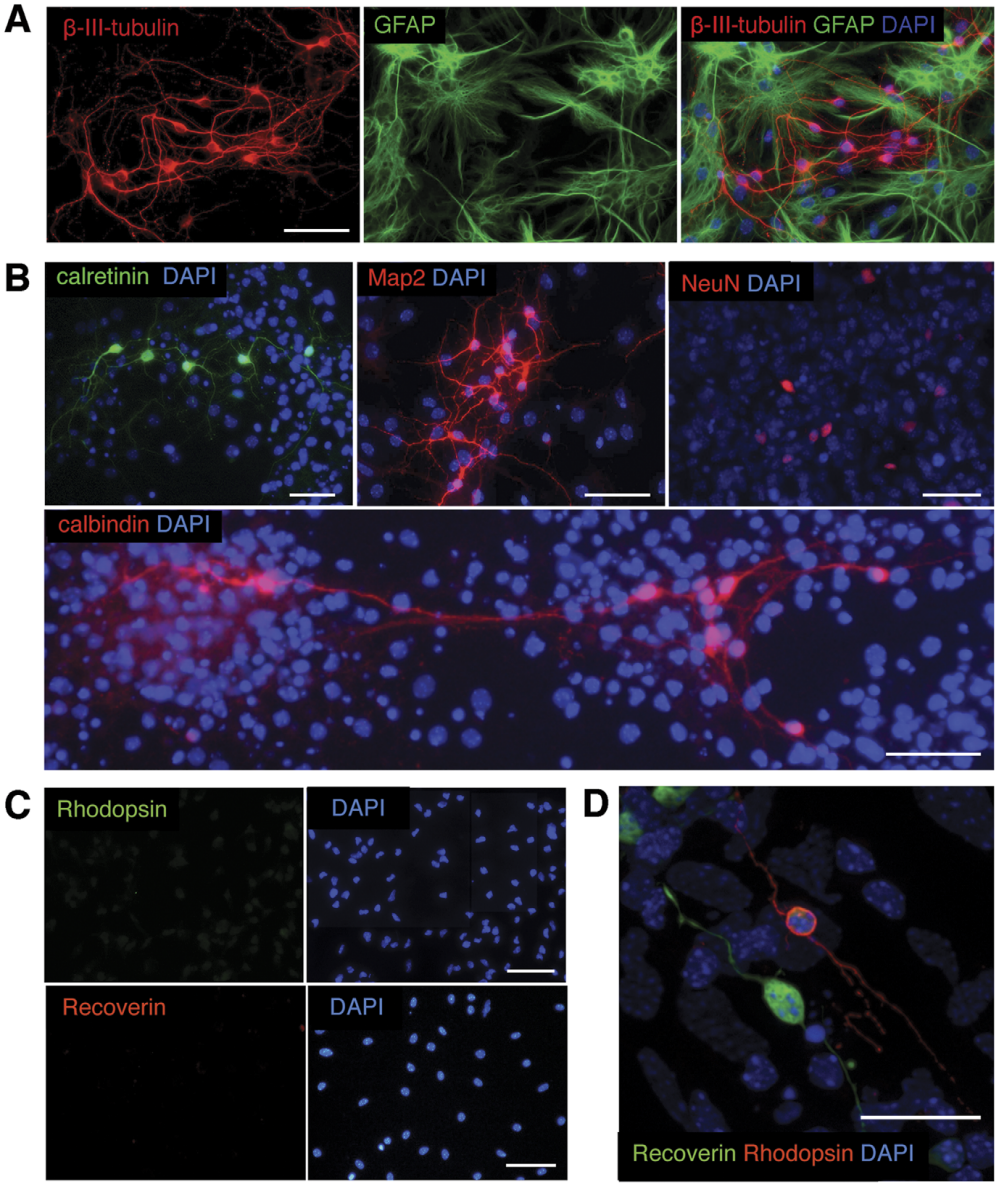
Differentiation of ‘RSCs’ *in vitro*. Following differentiation in 1% newborn calf serum expanded ‘RSCs’ showed immunoreactivity for the pan-neuronal markers β-III-tubulin (A, red) and MAP2 (B, red) or the glial marker GFAP (A, green). A subfraction of cells expressed the interneuron markers calretinin (B, green) or calbindin (B, red) or, after prolonged maintenance in differentiation conditions, the mature neuron marker NeuN (B, red). In contrast to primary neonatal retinal cells (D) subjected to the same differentiation conditions, expanded ‘RSC’ cultures are devoid of recoverin (red) or rhodopsin (green) expressing photoreceptors (C). Scale bars: 50 µm. Abbreviations: DAPI, 4,6-diamidino-2-phenylindole; GFAP, glial fibrillary acidic protein; MAP2, microtubule-associated protein 2; NeuN, neuron-specific nuclear antigen.

To induce the differentiation of ‘RSCs’, growth factors were removed from the culture medium and replaced by 1% NCS. After 10 days ‘RSCs’ were post-mitotic (i.e. Ki67 negative, data not shown) and most of the cells expressed either the astroglial marker GFAP or the pan-neuronal marker ß-III-tubulin (Figure 2A). Additionally, we occasionally observed few cells in long term cultures that were simultaneously immuno-positive for GFAP and ß-III-tubulin (data not shown). We further investigated the differentiation of ‘RSCs’ along the neuronal lineage and examined the expression of more mature neuronal markers. Some cells expressed MAP2, a marker of mature neurons, or the inter-neuron markers for the calcium binding proteins calretinin or calbindin (Figure 2B). A few cells also expressed the mature neuron marker NeuN (Figure 2B), however, NeuN expression was only detectable following prolonged differentiation times for up to 3 weeks. Although these proteins are expressed in specific neuronal cell types of the retina, they can also be found outside the retina in different neuronal cell types throughout the nervous system [32]–[37].

One neuronal cell type that is unique in terms of function and gene set in the retina are photoreceptors, which express proteins essential for the photo-transduction cascade. Indeed, a sub-fraction of primary cells isolated from the neonatal retina and cultured in differentiation medium without prior passaging expressed the photoreceptor-specific markers recoverin and rhodopsin and developed a morphology characteristic for photoreceptors *in vitro* with a small, round cell body and long, thin processes (Figure 2D). However, when passaged ‘RSCs’ were subjected to differentiation, expression of photoreceptor markers was no longer detectable (Figure 2C).

### Increased Neurogenic Differentiation by ‘Priming’ and Inhibition of Notch Signaling

**Figure 3:**
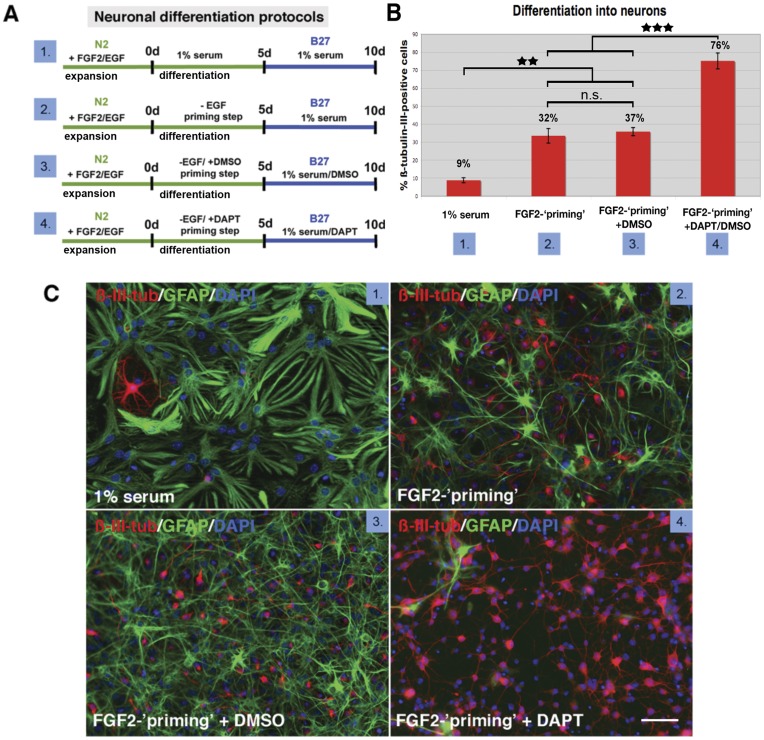
Increased neuronal differentiation of ‘RSCs’ by priming and notch inhibition. Generation efficiency of neuronal cell types by expanded ’RSCs’ is dependent on differentiation conditions. ’RSC’ cultures subjected to different differentiation conditions detailed in (A) and immunolabeled with antibodies directed against GFAP (C, green) and β-III-tubulin (C, red) contain different percentages of neurons in dependence of the applied protocol (B, C). The percentage of β-III-tubulin -positive neurons is significantly increased by ’neuronal-priming’ from <17% to <32% when compared to differentiation conditions where mitogens are replaced by NCS and can be further increased to <76% by inhibition of notch-signaling using DAPT (B). DMSO represents the DAPT control experiment with neuron numbers (<37%) corresponding to ’priming’ (B, C). Scale bars: 50 µm. Abbreviations: d, days; DAPI, 4,6-diamidino-2-phenylindole; DAPT, [N-(3,5-difluorophenacetyl)-L-alanyl]-S-phenylglycine t-butylester; DMSO, dimethyl sulfoxide; GFAP, glial fibrillary acidic protein. **p<0.01, ***p<0.001.

**Figure 4:**
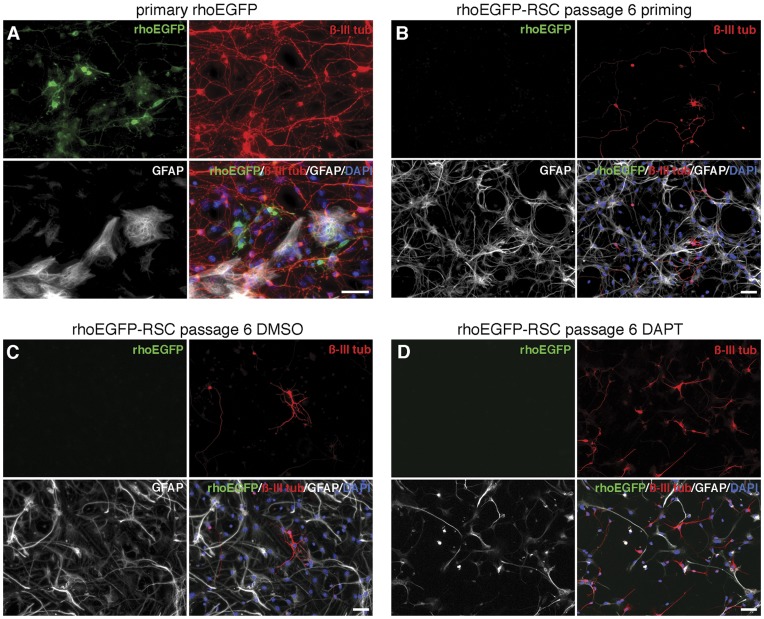
Differentiation of primary retinal cells and *in vitro* expanded ‘RSCs’ isolated from rhoEGFP reporter mice. Primary cells isolated at PN2 from retinas of *rhoEGFP* transgenic mice and cultured for 7 days *in vitro* (A) showed expression of GFP (green - identifying rod photoreceptors), β-III-tubulin (red - identifying neurons), or GFAP (white - identifying glial cells; merged images additionally contain nuclear DAPI staining). ‘RSCs’ generated from *rhoEGFP* transgenic mice and propagated *in vitro* for 6 passages were subjected to priming (B) or Notch inhibition with DAPT (D; C represents the DMSO control) differentiation conditions for 10 days. ‘RSCs’ differentiated into β-III-tubulin-positive neurons (red) and GFAP-positive glia (white), but did not show GFP expression indicative for photoreceptor differentiation (B, C, D). Scale bars: 20 um.

Recent studies suggested that the step-wise reduction of growth factors (‘priming’) from NSC or ’RSC’ cultures leads to increased neuronal or photoreceptor differentiation [22], [38]. Here we show that such priming-step resulted in a significantly increased number of ‘RSC’-derived ß-III-tubulin - positive cells (32%) when compared to differentiation with serum alone (17% ß-III-tubulin positive; Figure 3A–C). We further investigated the potential of *in vitro* expanded ‘RSCs’ to differentiate into neuronal phenotypes by inhibition of the Notch signaling pathway. Notch signaling plays important roles in the development of the CNS and retina and has a strong influence on cell fate decisions of NSCs and retinal progenitors [39]–[42]. Therefore the γ-secretase inhibitor DAPT, a potent inhibitor of notch activity, was added to the medium during primed ‘RSC’ differentiation. DAPT treated ‘RSCs’ showed a significantly increased number of ß-III-tubulin-positive cells (76%) in comparison to only primed (+ DMSO; 37%) or serum differentiated cells (17%, see above). However, expression of photoreceptor-specific markers like rhodopsin or recoverin or up-regulation of EGFP in photoreceptor-specific reporter *rhoEGFP*-‘RSCs’ could not be observed neither following priming nor notch inhibition (Figure 4 and not shown).

### Upon Transplantation Undifferentiated ‘RSCs’ Generate Glial and Neuronal Cell-types

To investigate the differentiation of *in vitro* expanded ‘RSCs’ *in vivo*, EGFP expressing donor cells (*actin-EGFP*-‘RSCs’) between passage 3–30 were transplanted into 28 wild-type C57BL/6J adult retinas. Of these, 20 retinas showed integration of reporter expressing donor cells and were further investigated.

**Figure 5:**
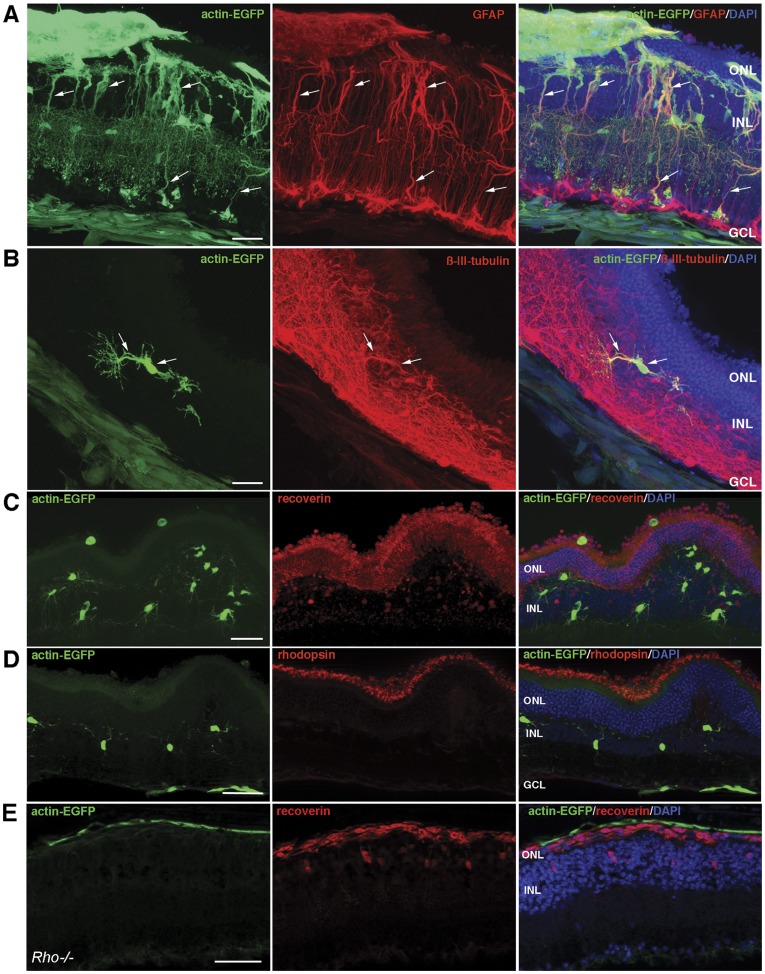
Transplantation of ‘RSCs’ into adult mouse retina. Immunohistochemical analysis on adult wild-type retinas transplanted with GFP-positive ‘RSCs’ (green in A, B, C, and D) revealed that the majority of integrated donor cells expressed GFAP, indicative for extensive glial differentiation (A, arrows). While some transplanted ‘RSCs’ differentiated along the neuronal lineage and expressed β-III-tubulin (B, arrows), grafted cells did not show recoverin (C) or rhodopsin (D) immunoreactivity. Also following transplantation into the degenerative retina of rho−/− mice (E) donor ‘RSCs’ (green) did not show immunoreactivity for recoverin (red). Scale bars: 50 µm. Abbreviations: DAPI, 4,6-diamidino-2-phenylindole; GCL, ganglion cell layer; GFAP, glial fibrillary acidic protein; INL, inner nuclear layer; IPL, inner plexiform layer; ONL, outer nuclear layer.

Following injection clusters of donor cells, identified by EGFP expression, were found in the vitreous or sub-retinal space. Some donor cells migrated into the host tissue (Figure 5) where they preferably incorporated into the ganglion cell layer (GCL), inner plexiform layer (IPL) and inner nuclear layer (INL). Most of the donor cells developed into GFAP-positive glial cells (Figure 5A) and few into β-III-tubulin expressing neurons (Figure 5B). Interestingly, grafted ‘RSCs’ did not integrate deeper than the INL, i.e. donor cells were not migrating into the ONL even when donor cell clusters were located in the subretinal space. From the subretinal location ‘RSCs’ sometimes extended processes through the ONL, but cell bodies of grafted cells were only rarely found in this retinal layer (Figure 5A). Immunohistochemistry using photoreceptor-specific antibodies against recoverin (Figure 5C) or rhodopsin (Figure 5D) showed no co-staining with EGFP and thus revealed that ‘RSCs’ did not differentiate along the photoreceptor lineage following transplantation into the wild-type retina (Figure 5C, D). Adult wild-type retinas might not provide a permissive environment for the generation of mature photoreceptors from stem/progenitor cells. Therefore, expanded ‘RSCs’ were additionally transplanted into the subretinal space of two retinal degeneration models characterized by photoreceptor loss, i.e. rhodopsin-deficient (rho−/−; [88]) *and P347S* [89] mice. Also in such diseased retinas expression of photoreceptor-specific markers by donor ‘RSCs’ could not be detected (Figure 5E and data not shown).

### *In vitro* Expanded ‘RSCs’ Acquire the Potential to Differentiate along the Oligodendrocyte Lineage

**Figure 6:**
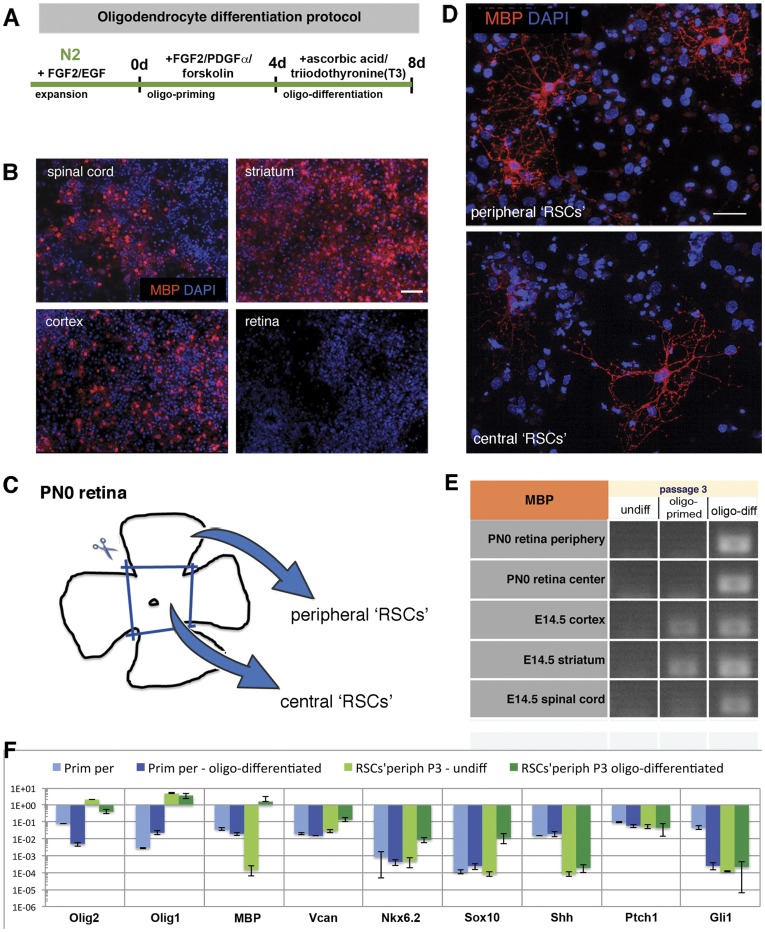
Oligodendrocyte differentiation of ‘RSCs’ *in vitro*. Whereas primary cells isolated at E14.5 from different regions of the CNS, i.e. spinal cord, striatum, and cortex, showed strong MBP expression (B) when subjected to the oligodendroglial differentiation protocol detailed in (A), primary retinal cells remained MBP-negative (B). However, after *in vitro* expansion central and peripheral ‘RSC’ (P3) cultures generated by appropriate dissection procedures (C) responded to the oligodendrocyte differentiation protocol with expression of MBP as shown by immunocytochemistry (D) and RT-PCR performed on RNA isolated from undifferentiated, oligo-primed and oligo-differentiated ‘RSCs’ and NSCs from P3 (E). Expanded peripheral ‘RSCs’ (P3) as well as their primary counterparts (peripheral retinal cells from PN0) were subjected to the oligo-differentiation protocol *in vitro* and their gene expression profile was investigated in detail using Q-PCR array for oligodendrocyte-related gene expression (F). Primary cells expressed moderate levels of *Olig1/2* genes, whereas expanded ‘RSCs’ contained very high levels of these transcripts. While primary cells subjected to oligo-differentiation conditions did not respond to the treatment with an increase in the expression of oligodendrocyte-related genes, expanded ‘RSCs’ exhibited a significant increase in *MBP* transcript levels along with other oligodendrocyte-related genes that were analyzed (*Vcan*, *Nkx6.2*, *Sox10*). Generation of oligodendrocytes from expanded ‘RSCs’ was Shh-independent since we could not detect endogenous *Shh* nor its downstream target *Gli1* (F). Scale bars: 100 µm (B) and 50 µm (D). Abbreviations: DAPI, 4,6-diamidino-2-phenylindole; E, embryonic day; MBP, myelin basic protein; Oligo1/1+2, oligodendroglial differentiation step 1/1+2; PN, postnatal day; T3, 3,3,5-triodothyronine; Rho, rhodopsin; Rxrg, retinoid X receptor gamma; Rcvrn - recoverin.

To evaluate if mitogen expanded ‘RSCs’ exhibit features of NSCs we investigated their potential to generate oligodendrocytes *in vitro* by subjecting the cells to culture conditions known to favor the differentiation of NSCs into oligodendrocytes [43]. This protocol involves, in a first step, a four day cultivation with FGF-2, PDGF and forskolin (‘oligo-priming’ step) followed by four day incubation with thyroid hormone T3 and ascorbic acid (‘oligo-differentiation’ step; Figure 6A). First we investigated how primary cells, i.e. not passaged, isolated from the neonatal retina adapted to the procedure and compared them with primary cells isolated from the embryonic spinal cord, striatum or cortex. Whereas cultures generated from the three brain regions (i.e. spinal cord, striatum, cortex) showed many cells positive for the oligodendrocyte-specific markers MBP (Figure 6B) and MAG (data not shown), retinal cells, as expected, were negative for these markers (Figure 6B and not shown).

During retina preparation the whole optic nerve was discarded. Additionally, to minimize the possibility of contaminating the ‘RSC’ cultures with oligodendrocyte progenitor cells (OPCs) we separated peripheral, distant to the optic disc, and central retinal regions and used them to generate peripheral and central ‘RSCs’, respectively (Figure 6C). Such obtained peripheral and central ‘RSCs’ both showed responsiveness for oligodendroglial differentiation treatment generating MBP-immunoreactive cells (Figure 6D) with a morphology similar to oligodendrocytes *in vitro* derived from expanded NSCs (data not shown). RT-PCR performed on undifferentiated or oligodendroglial differentiated cells with MBP-specific primers confirmed the presence of *MBP* transcripts in differentiated ‘RSCs’, as it was also detected for differentiated NSCs (Figure 6D). Finally, we also subjected *in vitro* expanded ‘RSCs’ from 14.5 days old embryonic retinas to oligodendrocyte inducing conditions, a developmental stage at which oligodendrocyte progenitor cells have not yet infiltrated the developing optic nerve. Also these differentiated ‘RSCs’ showed expression of oligodendrocyte-specific markers at protein and RNA levels (data not shown).

To confirm that peripheral ‘RSC’-derived oligodendrocytes resemble oligodendrocytes at the molecular level, we performed more detailed oligodendrocyte-related gene examination (Figure 6F). *Olig1* and *Olig2* are bHLH transcription factors important for oligodendrocyte specification and maturation [44]. It was also reported that *Olig2* is expressed in developing retina, particularly in proliferating retinal progenitor cells [45]. Q-PCR performed on primary neonatal peripheral retinal cells revealed moderate levels of *Olig2*, which was around 28 fold higher than expression of *Olig1*. By contrast, undifferentiated ‘RSC’ cultures from P3 showed increased expression of these genes - up to 1859 fold increase of *Olig1* and up to 27 fold of *Olig2* (Figure 6F) when compared to their primary source cells. Moreover, following oligo-differentiation of ‘RSCs’ all analyzed oligodendrocyte-related genes were up-regulated (Figure 6F): *MBP* up to 14500 fold, *Vcan* - 7 fold, *Nkx6.2*–49 fold and *Sox10* - up to 191 fold. In contrast, Q-PCR analysis of primary peripheral cells showed low expression of *MBP* and *Vcan* and no detectable levels of *Nkx6.2* and *Sox10* - transcriptions factors crucial for oligodendrocyte maturation and myelination [46], [47]. Importantly, a similar increase in expression levels of these transcripts was not observed in primary retinal cells that were maintained in oligodendrocyte-differentiation conditions, indicating that the starting population of peripheral ‘RSCs’ was devoid of OPCs or other cells with the potential of oligodendrocyte differentiation. Due to the findings that expanded ‘RSCs’ were cultured in Shh-free conditions, contained very low levels of endogenous *Shh* and its downstream target *Gli1*, which remained unchanged upon oligo-differentiation (Figure 6F), we suggest that their conversion into cells able to acquire an oligodendroglial fate in the course of *in vitro* expansion was Shh-independent.

### ‘RSC’-derived Oligodendrocytes Myelinate Axons upon *in* *vivo* Transplantation

**Figure 7:**
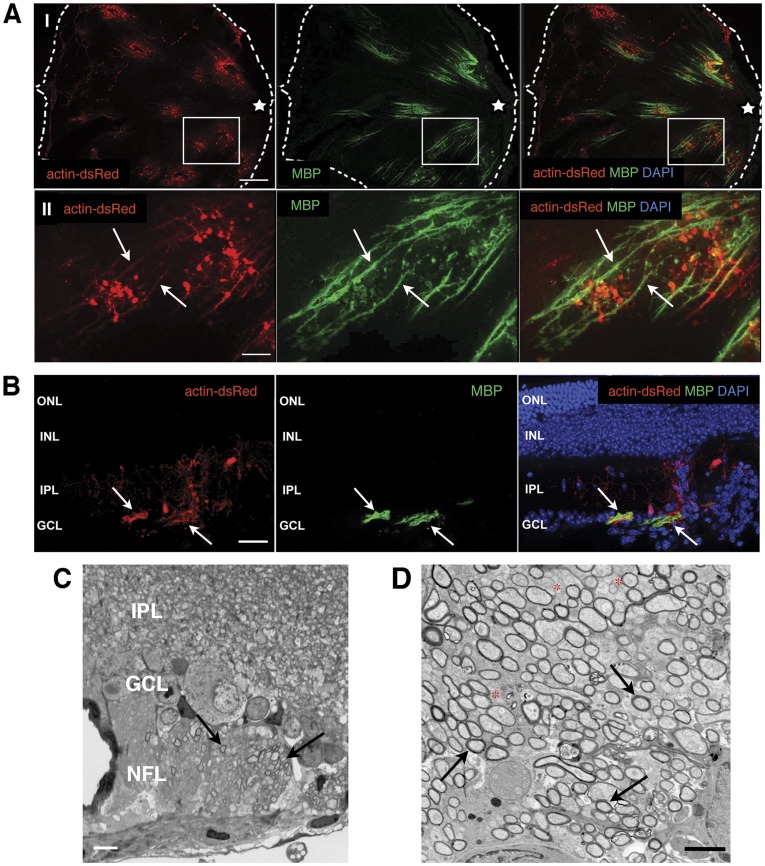
*In vivo* myelin-formation by pre-differentiated ‘RSCs’. Analysis of retinas four weeks after intraretinal transplantation of oligo-primed *actin-dsRed*-‘RSCs’ (red) into adult mice. Many donor cells identified by dsRed expression (A) were located on the vitreal side of the retina (the edges of a flat mounted retina are marked by the dashed white line) and some formed elongated structures radiating towards the optic disc (white star)(some are labeled by arrows in AII; AII is an enlarged view of the boxed area in AI) that are positive for MBP (green, A). Following transplantation of oligo-primed *actin-dsRed* expressing ‘RSCs’ (red, B), donor cells integrated into the GCL and IPL (red, B) of the host retina. Co-localization of MBP immunoreactivity (green, B) and dsRed fluorescence was restricted to the GCL (B, nuclear DAPI staining (blue) is additionally present in the merged image). Histological analysis of a semi-thin section revealed myelinated axons in the nerve fiber layer of an experimental retina (C; arrows). Transmission electron microscopy confirmed the presence of compact myelin around many RGC axons (some labeled by arrows) in retinas transplanted with ‘RSCs’ (D). Note the increased diameter of myelinated in comparison to unmyelinated axons (D; some labeled by red stars). Scale bars: 200 µm (AI), 50 µm (AII, B), 10 µm (C), 2500 nm (D). Abbreviations: DAPI, 4,6-diamidino-2-phenylindole; GCL, ganglion cell layer; INL, inner nuclear layer; IPL, inner plexiform layer; ONL, outer nuclear layer.

**Figure 8:**
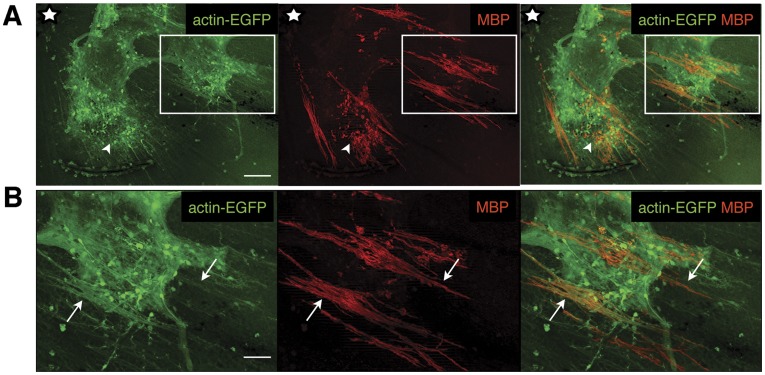
*In vivo* myelin-formation by pre-differentiated ‘RSCs’ derived from peripheral regions of the developing retina. Analysis of wild-type retinas four weeks after transplantation of oligo-primed *actin-EGFP*-‘RSCs’ (green) from P3 into adult mice. Many GFP-expressing donor cells form MBP-positive elongated structures on the vitreal side of the retina (red; some are labeled by arrows in B; B is an enlarged view of the boxed area in A) showing MBP-positive fibers radiating towards the optic disc (white star). Scale bars: 100 µm (A), 50 µm (B).

To evaluate if MBP-positive cells generated *in vitro* from expanded ‘RSCs’ can differentiate into fully mature myelinating oligodendrocytes, transplantation experiments were performed. The intra-retinal part of retinal ganglion cell (RGC) axons in the mouse eye is unmyelinated [29] but competent to become myelinated following transplantation of myelinogenic cells [48], [49]. Therefore, expanded (3, 10, 16, or 17 passages) ‘RSCs’ generated from reporter mice (*actin-dsRed* or *actin-EGFP*) were oligo-primed (i.e. maintained in medium containing FGF-2, PDGF and forskolin) and then transplanted into the vitreous of 40 adult wild-type eyes. 4 to 5 weeks post-grafting the recipient animals were sacrificed and eyes investigated for the presence of dsRed- or EGFP-expressing donor cells. All recipient retinas showed integrated donor cells and were further investigated. Analysis of flat mounted retinas transplanted with ‘RSCs’ derived from either whole retina (Figure 7) or peripheral retina (Figure 8) revealed that a portion of donor cells were located at the retinal surface where they formed cell clusters (Figure 7AI, 8) and long extensions (Figure 7AII, 8B, some labeled with arrows) radiating towards the optic nerve head (Figure 7AI, 8A labeled with a star). Immunohistochemical analysis showed co-labeling of donor derived extensions and MBP-reactivity, suggesting that transplanted donor cells had formed myelin sheaths around endogenous RGC axons (Figure 7A, 8). Indeed, further analysis of sectioned experimental retinas revealed that several donor cells had integrated into the GCL and IPL of host retinas (Figure 7B) and that donor-derived processes restricted to the GCL/nerve fiber layer were positive for MBP (Figure 7B, arrows). Importantly, ultra-structural investigation of regions containing donor cell-derived extensions showed the presence of myelin sheaths around RGC axons in host retinas (Figure 7C, D, arrows).

### Some Oligo-differentiated ‘RSCs’ Show Ectopic Expression of Photoreceptor Genes but Fail to Generate Photoreceptors Both *in vitro* and after Subretinal Transplantation

**Figure 9:**
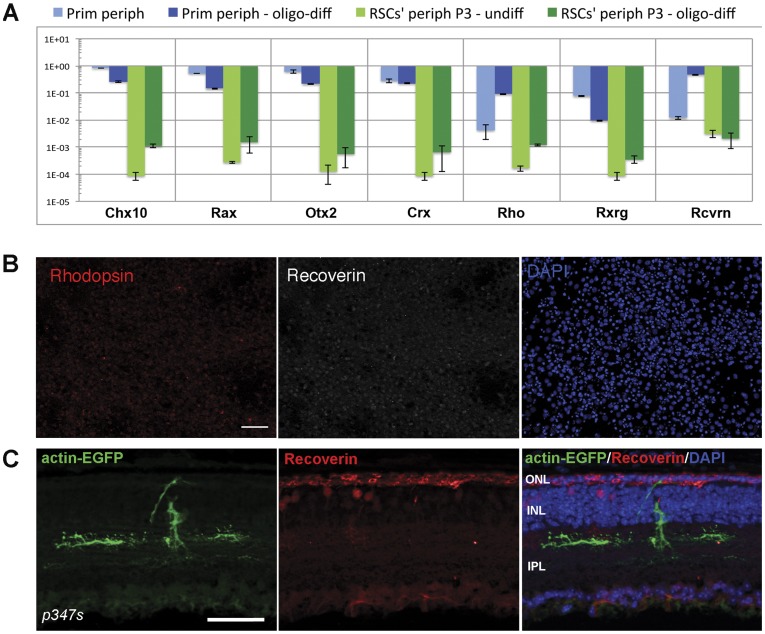
Oligo-differentiated ‘RSCs’ show slight elevated levels of photoreceptor-specific genes, but fail to generate photoreceptors. Cultivated peripheral ‘RSCs’ subjected to the full oligodendrocyte differentiation protocol showed a slight increase in the expression of the retina-specific genes *Chx10*, *Rax*, *Otx2*, *Crx*, *rhodopsin* and *RXRgamma* as analysed by Q-PCR albeit at very low absolute levels (A). Immunocytochemical analysis of ‘RSCs’ subjected to oligodendrocyte differentiation *in vitro* did not reveal positive signals for rhodopsin (red) or recoverin (white) proteins and therefore no evidence for generation of photoreceptors (B). Also following transplantation of oligo-primed ‘RSCs’ (C; green) into the subretinal space of degenerative P347S mice (C) donor cells (green) did not show immunopositivity for the photoreceptor marker recoverin (red). Nuclear DAPI staining is shown in blue, rhodopsin localization in red, recoverin localization in white (B). Scale bars: 50 µm (B). Abbreviations: DAPI, 4,6-diamidino-2-phenylindole.

Treatment of ‘RSCs’ from peripheral retinal regions with PDGF, forskolin, thyroid hormone and ascorbic acid (full oligodendrocyte-differentiation protocol) induced slight up-regulation of genes characteristic for the developing or mature retina as depicted by Q-PCR measurements (Figure 9A) including *Chx10*, *Rax*, postmitotic *Otx2* and *Crx* as well as *rhodopsin* and *RXRgamma*. However, absolute expression levels of all analyzed genes were very low. Interestingly, other common photoreceptor specific genes (e.g. *recoverin*) did not show changes in expression levels (Figure 9A) suggesting an ectopic expression of such retina-related genes rather than reflecting a retinal or photoreceptor phenotype. To further examine if such minimal changes of gene expression reflected generation of photoreceptors, we subjected the *in vitro* oligodendrocyte-differentiated ‘RSCs’ to immunocytochemical analysis using anti-rhodopsin and anti-recoverin antibodies (Figure 9B). However, immuno-positivity for photoreceptor markers could not be detected (Figure 9B). Furthermore, the differentiation potential of oligo-predifferentiated ‘RSCs’ was investigated *in vivo* following transplantation into the subretinal space of mice as this retinal location provides a permissive environment for maturation of grafted cells into photoreceptors both in wild-type [9], [11] and retinal degeneration (P347S mouse: Eberle D, Kurth T, Santos-Terreira T, Wilson J, Corbeil D, Ader M – unpublished observations) mice. However, oligo-predifferentiated *actin-EGFP* ‘RSCs’ injected to the subretinal space of wild type and photoreceptor degeneration mice revealed poor integration and survival and no evidence for differentiation along the photoreceptor lineage (Figure 9C and data not shown).

## Discussion

*In vitro* propagation of stem and progenitor cells from different regions of the CNS has initiated investigations into their possible use to replace lost cells in several neurodegenerative diseases [50]–[52]. *In vitro* expandable cells isolated from the mammalian retina, termed ‘RSCs’, are discussed as candidates in retinal degenerative conditions characterized by photoreceptor loss [53].

During development multipotent retinal progenitors move through several competence states thereby producing in a timely fashion six neuronal (RGCs, amacrines, bipolar, horizontal, rods, cones) and one glial (Müller cells) cell types [28], [54], [55]. Efficient *in vitro* expansion protocols for these multipotent retinal progenitors would be a prerequisite to use such cells in therapeutic cell replacement applications. Our study reveals that ’RSCs’ isolated from the developing mouse retina are highly expandable *in vitro* as monolayers in the presence of FGF-2 and EGF and capable to give rise to neuronal and glial subtypes. Furthermore, they respond to Notch signaling inhibition with increased generation of neurons. However, ‘RSCs’ loose features of primary retinal progenitors including expression of retinal progenitor transcription factors and the potential to generate photoreceptors. Furthermore, we report for the first time that *in vitro* expanded ‘RSCs’, unlike primary retinal cells, can differentiate into oligodendrocytes *in vitro* that extensively myelinate retinal ganglion cell axons after intraretinal transplantation *in vivo*.

For the development of a cell-based strategy to treat retinopathies such as in RP or AMD it is necessary to find a proper cell source for replacing degenerated photoreceptors. Induced pluripotent stem (iPS) cells, embryonic stem (ES) cells [56]–[59], NSCs, or ‘RSCs’ represent currently the most favorable cell populations for such applications. Whereas the generation of photoreceptors from iPS- and ES cells has been demonstrated in several studies [57], [59]–[61], NSCs never gave rise to photoreceptors [48], [49], [62], [63]. To evaluate a more retina-specific cell source, several studies reported the isolation and *in vitro* expansion of ‘RSCs’ from the developing mammalian retina that showed multipotency and the capacity to form photoreceptors *in vitro* and following transplantation *in vivo* [21]–[23], [64], [65]. However, evidence for photoreceptor generation was based on immunochemistry and/or RT-PCR analyses alone and the morphology of ‘RSC’-derived photoreceptors in these studies did not resemble the specific architecture of mature photoreceptors following transplantation, as it was demonstrated for primary photoreceptor precursors isolated from photoreceptor-specific reporter mice (i.e. small, round cell bodies located in the ONL, the formation of inner and outer segments, and generation of axonal terminals in close proximity to bipolar cells in the OPL) [8], [9], [11], [12]. These findings indicate that *in vitro* expanded ‘RSCs’, in contrast to committed photoreceptor precursors, are incapable to give rise to mature photoreceptor cells after transplantation. The failure of ‘RSCs’ to differentiate into true photoreceptors might be either due to the non-permissive environment of the adult retina to induce neuronal differentiation or due to a principle intrinsic incapability of expanded ‘RSCs’ to differentiate along the photoreceptor lineage. Indeed, a study by Mansergh et al. [66] reported rapid decrease of *rhodopsin* expression levels after subjecting retinal cells isolated from newborn mouse retinas to expansion culture conditions. The remaining *in vitro* expanded ’RSCs’ were shown to have a similar expression pattern to undifferentiated neural cells and treatment with retinoid acid (RA) did not induce reappearance of *rhodopsin* expression [66], despite prior evidence that in dissociated retinal cells, retinal explants or differentiating mouse ES cell cultures RA can induce photoreceptor marker expression [61], [67], [68]. Additionally, Gamm et al. [69] reported that human retinal progenitor cells isolated during development and expanded in growth factor containing- and RPE conditioned medium lost neurogenic, including photoreceptor, differentiation potential over time resulting in neurospheres restricted to a glial fate [69]. Our data indicate that ’RSCs’ indeed have the potential to generate neuronal and glial cell types and that inhibition of Notch signaling during differentiation enhances the efficacy of neuronal differentiation to 76%, as it was also reported for NSCs [42], [70]. However, ‘RSCs’ expanded *in vitro* under conditions described in this study failed to generate photoreceptors both *in vitro* and *in vivo* following transplantation into wild-type and retinal degeneration mice. The finding that *in vitro* passaged ‘RSCs’ fail to generate photoreceptors is in obvious contrast to published data [21]–[24] and we are suggesting that the unphysiological high concentrations of FGF2 and EGF in the culture media might be a reason for the loss of retinal identity of *in vitro* expanded ‘RSCs’ (see also below). However, we can not rule out that subtle differences in culture conditions of ‘RSCs’ used in other studies - e.g. cultivation of cells as neurospheres or under adherent conditions, differences in dissociation procedures, or differences in medium compositions and concentrations of FGF2/EGF – have profound effects on the differentiation potential of such *in vitro* propagated retinal cells.

‘RSCs’ expressed markers characteristic for CNS stem/progenitor cells like *nestin*, *Sox2* and *Pax6* that also play essential roles during retina formation and retinal progenitor propagation *in vivo* [27], [71], [72]. Interestingly, following passaging expression of transcription factors indispensable for retina formation such as *Rax* and *Chx10* [73], [74] dropped rapidly. *Lhx2* is a LIM-homeobox transcription factor, which has been suggested to be crucial in establishing primitive retinal identity [75], controls expression of *Rax*, *Six3*, and *Pax6* and its interactions with the latter may directly regulate expression of *Six6*. We observed that *Lhx2*, *Six3* and *Six6* showed a tendency to be down-regulated to a variable extent in ‘RSCs’ at higher passages. In line with our observations, Schmitt et al. [76] showed, using customized microarrays, that *in vitro* expanded human retinal progenitor cells also down regulate the expression of genes important for retinal development like *Chx10*, *Lhx2*, and *Six3*. Therefore, the concomitant down-regulation of factors highly important in retina development and retinal progenitor cell maintenance like *Rax*, *Chx10*, *Lhx2*, and *Six6* with sustained expression of more common neural stem/progenitor markers like *nestin*, *Sox2*, *Pax6* and Notch pathway components let us conclude that expanded ‘RSCs’ loose their regional identity over time and acquire features similar to *in vitro* expanded NSCs.

Mouse retina is devoid of endogenous oligodendrocytes and myelin. In the developing mouse eye oligodendrocyte-progenitor cells (OPCs) are prevented from migrating into the retina from the optic nerve [29], [30], [77] and primary retinal progenitor cells do not adopt oligodendroglial fate neither during retina development [27], [28] nor *in vitro*, when subjected to culture conditions that promote oligodendrocyte formation [29] (Figure 6). Therefore surprisingly, when ‘RSCs’ were subjected to the oligodendrocyte differentiation protocol, we observed the generation of MBP and MAG-positive oligodendrocytes. Furthermore, when pretreated with PDGF and forskolin alone and transplanted to the vitreal side of the retina, ‘RSCs’ formed MBP-immunoreactive elongated structures, which were reminiscent of myelinated RGC axon fascicles described after transplantation of NSCs into the mouse retina [48], [49]. MBP reactivity was indeed confined to the nerve fiber-(NFL) and ganglion cell layer (GCL). Moreover, ultrastructural analysis revealed that many axons within the NFL and GCL were extensively surrounded by myelin sheaths.

*In vivo*, stem cells reside in specific cellular micro-environments that support self-renewal and regulate the balance between proliferation and differentiation [78], [79]. However, *in vitro* propagation of neural and retinal stem cells is commonly performed in unphysiological, mitogen-rich conditions that do not recapitulate conditions present *in vivo*. It was shown previously that mitogens influence the transcriptional and cellular properties of NSCs [31], [80] and recent studies indicate that *in vitro* FGF-2 treatment induce generation of oligodendrocytes in dorsally derived embryonic cerebral cortical cells [81], [82] and in dorsal spinal cord cells via a Shh-dependent- [16] or independent manner increasing the expression of *Olig1*, *Olig2* and Nkx2.2 genes [83]. Based on these findings and the observations presented here we speculate that generation of myelinating oligodendrocytes by expanded cells derived from the developing retina, might result from an exposure to high concentrations of FGF-2, a mitogen commonly used for stem cell propagation. Since primary retinal progenitors represent a highly heterogenous cell population [84], it also cannot be excluded that the developing retina contains a progenitor subpopulation with specific responsiveness to the used expansion conditions that never gives rise to photoreceptors. Such hypothetical retinal progenitor subpopulation might have the competence to develop along the oligodendrocyte lineage upon *in vitro* expansion. Further detailed lineage fate analysis of single cells is necessary to unravel the exact source from which *in vitro* expanded ‘RSCs’ derive.

Taken together, we suggest that the *in vitro* expanded ‘RSCs’ described here do not resemble a defined cell-type that does exist *in vivo* but rather represent a ‘synthetic’ stem cell state that is induced by the specific culture conditions as it is also discussed for *in vitro* expanded NSCs [18], [19].

From a therapeutic point of view, ‘RSCs’ incompetent to generate photoreceptors but competent to generate oligodendrocytes do not represent a useful candidate cell population for retinal replacement therapies. Myelination of intraretinal segments of RGC axons, which occurs in 1% of the human population, leads to decreased vision acuity and visual field defects [85]. Thus, our results indicate that the culture conditions for retina-derived cells do not support the expansion of ‘true’ retinal progenitor cells. Therefore, it is crucial to use/develop expansion methods which include optimized extrinsic factors in combination with stringent protocols that sustain the expression of retina-specific genes in expanded ‘RSCs’ for maintaining the capacity to differentiate along the photoreceptor lineage as it was e.g. recently shown in a 3D culturing system for ES cells [59].

In conclusion, *in vitro* expanded stem cell cultures generated from the developing retina have been widely used for *in vitro* experiments and transplantation studies and are discussed as promising cell populations to replace lost photoreceptors by cell transplantation. Our data implement that retina-derived cells subjected to FGF-2/EGF growth conditions represent multipotent stem cells that can generate neuronal and glial phenotypes. However, these cells lost typical retinal progenitor characteristics during expansion, and therefore failed to form photoreceptors. Instead, a fraction of expanded ‘RSCs’ could be differentiated into myelin forming oligodendrocytes. The presented results imply the importance of rigorous comparison of primary stem/progenitor cells and their cultured counterparts.

## Materials and Methods

### Animals

Retinal cells, which upon in vitro expansion are termed ‘retinal stem cells’ (‘RSCs’) as suggested in previous studies [21], [22] were generated from embryonic day (E) 14.5 or neonatal (postnatal day (PN) 0–1) wild-type (C57BL/6J), actin-EGFP [86], actin-dsRed (The Jackson Laboratory, Maine, USA) or rhodopsinEGFP (rhoEGFP) [87] reporter mice. In actin-EGFP and actin-dsRed transgenic mice the corresponding reporter is driven by the ubiquitously active chicken beta actin promoter coupled with the cytomegalovirus (CMV) immediate early enhancer. RhoEGFP mice were generated by knocking in the human rhodopsin gene fused to EGFP into the mouse rhodopsin locus thereby restricting EGFP expression to rod photoreceptors. NSCs were isolated from E14.5 wild-type embryos (C57BL/6J).

*In vivo* transplantation experiments were performed into either wild-type C57BL/6J (8–22 weeks old; n = 68) or retinal degeneration, i.e. 9 weeks old rhodopsin-deficient (Rho−/−; n = 4; [88]) and 9 weeks old P347S (n = 12; [89]) recipient mice. Following transplantation the animals were allowed to survive for 2–6 weeks.

All animal experiments were approved by the ethics committee of the TU Dresden and licenses for removal of organs and transplantation of cells into the retina were provided by the Landesdirektion Dresden (Az.: 24D-9168.24-1/2007-27 and Az.: 24D-9168.11-1/2008-33).

### Isolation and Culturing of ’RSCs’ and NSCs

‘RSC’ cultures were generated with some modifications as described elsewhere [21], [22], [66]. Briefly, following decapitation and removal of the eyes donor retinas were separated from the retinal pigmented epithelium, optic nerve/disc, pigmented ciliary margin and lens in cold, sterile Hank’s Buffered Salt Solution (HBSS; Invitrogen, Darmstadt, Germany). Some of the retinas were additionally intersected to separate peripheral (the outer most third) and central regions. The retinas were digested at 37°C in 0.05% trypsin solution (Sigma-Aldrich, Munich, Germany) and after 20 min the reaction was stopped by adding a mixture of trypsin inhibitor (Roche, Mannheim, Germany) and DNase I (Sigma-Aldrich) at a final concentration of 2 mg/ml and 100 µg/ml, respectively. After trituration with a fire-polished pasteur pipette the cells were centrifuged for 7 minutes at 1400 rpm and the pellet resuspended in basic medium (Dulbecco’s modified Eagle’s medium (DMEM)-F12 containing L-Glutamine, 15 mM Hepes, 1,2 g/l NaHCO (PAN Biotech GmbH, Aidenbach, Germany) supplemented with 1% N2 supplement (Invitrogen) and Penicillin-Streptomycin solution (1∶100; Invitrogen). The cells were subsequently counted with a haemocytometer and seeded at a density of 250,000 cells/cm^2^ in culture medium containing 20 ng/ml EGF (PeproTech GmbH, Hamburg, Germany) and 20 ng/ml FGF-2 (Miltenyi Biotec, Bergisch Gladbach, Germany). The culture medium was exchanged on alternate days. First passage was carried out around 3 weeks after seeding whereas following passages were performed every 4 days. For detachment of cultured cells Accutase (PAA Laboratories, Pasching, Austria) was used. The cells were afterwards seeded at a density of 50,000 cells/cm^2^. NSCs were isolated from E14.5 wild type mouse embryos, initially expanded as neurospheres and subsequently converted into adherent NS cells as described previously [15]. Briefly, cortical, striatal and spinal cord tissues were digested in 0.05% trypsin solution, triturated, span down and subsequently seeded onto 0.1% gelatin-coated cell culture dishes in NS-A medium (Biozol, Eching, Germany) containing 10 ng/ml EGF and 10 ng/ml FGF-2. Passaging was performed according to the protocol detailed for ‘RSCs’ (see above).

### Differentiation of ‘RSCs’ *in vitro*

For standard differentiation ‘RSCs’ growing on PLL- and laminin-coated coverslips were kept for 10 days in basic medium in which growth factors were replaced by 1% newborn calf serum (NCS) and 1% N2 supplement was switched after 5 days to 2% B27 supplement. For increased neuronal differentiation ‘RSCs’ were subjected to a 2-step differentiation protocol involving ‘priming’ - a stepwise growth factor withdrawal, as described previously [22], [38]. In brief, cells were cultured first for 5 days in a medium containing FGF-2 alone (no EGF), followed by another 5 days replacing FGF-2 with 1% NCS and 1% N2 with 2% B27 supplement. In parallel, cells were treated according to the same protocol but in the additional presence of 10 µM [N-(3,5-difluorophenacetyl)-L-alanyl]-S-phenylglycine t-butylester (DAPT; Sigma-Aldrich), a notch pathway inhibitor, or DMSO (Roth, Karlsruhe, Germany) as a control.

### Oligodendroglial Differentiation of ‘RSCs’ and NSCs

For differentiation along the oligodendroglial lineage ‘RSCs’ as well as NSCs were subjected to protocols described previously [43]. In brief, cells were plated on a gelatin- or matrigel- (diluted in basic medium in ratio 1∶3; BD Biosciences, Germany) coated surface and kept in culture medium containing FGF-2/EGF for 4 days. Cells were then propagated for 4 days in medium containing 10 ng/ml FGF-2, 10 ng/ml platelet-derived growth factor (PDGF; R&D Systems) and 10 µM forskolin (Sigma-Aldrich) (in the following termed ‘oligo-priming’ step), followed by 4 days of final differentiation (‘oligo-differentiation’ step) in a medium lacking mitogens but supplemented with 3,3,5-triodothyronine (T3; 30 µg/ml; Sigma-Aldrich) and ascorbic acid (200 µM, Sigma-Aldrich).

### RNA Isolation, RT-PCR and Q-PCR

Using the RNeasy Mini Kit (Quiagen, Hilden, Germany) total RNA was extracted from primary retinal tissue and ‘RSC’ or NSC cultures maintained either in an undifferentiated state or differentiated along the oligodendroglia cell lineage.

RT-PCR experiments were performed on undifferentiated, isolated from whole retina tissue, neonatal ‘RSCs’ from early and late passages (P3 and P20, respectively), and compared with NSC (P5 of striatal and P10 of spinal NSCs) and primary neonatal (PN0) and adult retina (20 weeks). 150 ng RNA of each sample was subjected to cDNA synthesis by using Oligo(dT) primers (Biomers, Ulm, Germany) and Superscript II Reverse Transcriptase (Invitrogen). PCRs from first-strand cDNA were performed using the MangoTaq DNA Polymerase (Bioline, Germany) and specific primers (see Table 1). All primer sets were tested and optimized for several different annealing temperatures (50–60°C) for 35 cycles.

**Table 1:** Table of used primers.

Primers		Sequence	Size (bp)	Annealing Temp (°C)
*nestin*	F	5′- CCTCAACCCTCACCACTCTATTTT -3′	337	61
	R	5′- GCTTTTTACTGTCCCCGAGTTCTC -3′		
*Sox2*	F	5′- AACCCCAAGATGCACAACTC -3′	202	55
	R	5′- CTCCGGGAAGCGTGTACTTA -3′		
*Pax6*	F	5′- CACCACACCTGTCTCCTCCT -3′	202	62
	R	5′- ATAACTCCGCCCATTCACTG -3′		
*Notch1*	F	5′- AGTCTGCAGGCTCCAGTGTT -3′	200	62
	R	5′- CCGTCTGTCCTCAGTTGGAT -3′		
*Hes1*	F	5′- AAGTCCCTAGCCCACCTCTC -3′	195	62
	R	5′- AGGCGCAATCCAATATGAAC -3′		
*Hes5*	F	5′- CACCGGGGGTTCTATGATATT -3′	180	60
	R	5′- CAGGCTGAGTGCTTTCCTATG -3′		
*Lhx2*	F	5′- TCAACTGCTTCACATGCACA -3′	195	58
	R	5′- AGCCCAATCCTGCACTCTTA -3′		
*Rax*	F	5′- CCCTGAGGCTAAACTTGCAG -3′	207	55
	R	5′- GTTCCCTTCTCCTCCTCCAC -3′		
*Chx10*	F	5′- CAATGCTGTGGCTTGCTTTA -3′	157	65
	R	5′- CTTGAGAGCCACTGGGCTAC -3′		
*Six3*	F	5′- GGGAGAAGGAGGCAGTTTTC -3′	200	62
	R	5′- TTTACCATGCAAACGAACCA -3′		
*Six6*	F	5′- TCCTGCGGGTATTTCACTTC -3′	204	55
	R	5′- TGGCTTCTTCACACAGAACG -3′		
â-actin	F	5′- CGTGGGCCGCCCTAGGCACCA -3′	243	62
	R	5′- TTGGCCTTAGGGTTCAGGGGG -3′		

Q-PCR was performed on RNA samples isolated from undifferentiated and fully oligodendrocyte-differentiated peripheral as well as central neonatal ‘RSCs’ from P3 and P10 along with their primary cell counterparts. PCR-array used for Q-PCR was provided by SABiosciences-Qiagen (USA). 200 ng of RNA of each sample was processed according to manufacturer’s instructions. Briefly, genomic DNA elimination and RT step were performed with RT^2^ First Strand Kit (SABiosciences - Qiagen). Such obtained cDNA of test (oligodendrocyte-differentiated) and control (undifferentiated) samples was mixed with RT^2^ qPCR Master mix and distributed across 96-well PCR array plates containing lyofilized probes for custom (eye field transcription factors, genes expressed in mature retinal neurons, genes related to oligodendrocyte cell development, components of certain cellular cell pathways) and housekeeping genes as well as controls required for normalization of data. After cycling with Q-PCR System M×3000P (Agilent, Boeblingen, Germany) the data was analyzed with SABiosciences-provided software. Gene expression levels are related to the average expression levels of housekeeping genes. The experiment was performed in duplicate for primary cells or in triplicate, i.e. for 3 independent ‘RSC’ cultures derived from either central or peripheral retina regions.

### Transplantation into the Mouse Retina

For transplantation studies undifferentiated or oligo-primed ‘RSCs’ of passages 3–30 were used. Subretinal transplantations were performed to study ability of ‘RSCs’ to integrate into the outer nuclear layer (ONL) and form photoreceptors. Intravitreal transplantations were done to analyze the potential of donor cells to form myelin sheaths around retinal ganglion cell (RGC) axons.

Prior to transplantation recipient mice were deeply anesthetized with an intraperitoneal injection of ketamine (0.75 mg/10g body weight, Ratiopharm, Ulm, Germany) and medetomidine hydrochloride (0.01 mg/10g body weight; Dormitor, Pfizer, Berlin, Germany) and pupils were dilated with topical administration of 2.5% phenylephrin (TU Dresden pharmacy, Germany) and 1% tropicamid (Mydrum, Dr Mann Pharma GmbH, Berlin, Germany). Using a 34 gauge needle connected to a Hamilton syringe 2 µl suspension containing 400,000 cells were injected intravitreally or subretinally with gently lesioning of the retina as described in detail before [9], [48], [49]. For recovery experimental animals received an intraperitoneal injection of atipamozole hydrochloride (0.1 mg/10g bodyweight, Antisedan, Pfizer) for reversal of medetomidine. Experimental animals were sacrificed 2 to 6 weeks following transplantation.

### Immunocytochemistry and Immunohistochemistry

For immunocytochemistry primary cells isolated from the neonatal retina and embryonic cortex, striatum and spinal cord as well as cultured cells were seeded onto PLL- and laminin- or matrigel-coated coverslips and subjected to one of the differentiation protocols. Subsequently the cells were fixed with 4% paraformaldehyde (PFA) in PBS for 15 min at room temperature (RT), washed 3x 5 min in PBS and rinsed for 30 min in blocking solution containing 5% goat serum (GS; Sigma-Aldrich), 1% bovine serum albumine (BSA; Serva, Austria) and 0.3% Triton X-100 (TU-Dresden pharmacy), followed by incubation with primary antibodies (1.5 h, then rinsed 3×5 min with PBS) and secondary antibodies for additional 1 h. Cells were subsequently counter stained with DAPI solution (1∶20,000; Sigma-Aldrich). After washing with PBS (3×5 min) coverslips were embedded in Aqua-Poly/Mount (Polysciences Inc., Eppelheim, Germany) on glass slides.

Transplanted animals were perfusion fixed with 4% PFA and eyes were postfixed for additional 12 h. Dissected retinas were either embedded in 4% agarose and sectioned with a vibrating microtome (Leica Microsystems, Germany) into 30 µm thick slices or flat mounted and processed for immunohistochemistry. Immunostaining of retinal tissue was performed as described above for cells but with prolonged incubation times for primary antibodies (12 h at 4°C).

The following antibodies were used: mouse anti-calbindin (1∶10,000, Swant; Marly, Switzerland), rabbit anti-calretinin (1∶5000; Swant), mouse anti-glial fibrillary acidic protein (GFAP; 1∶500; Sigma-Aldrich), rabbit anti-GFAP (1∶500; DAKO, Hamburg, Germany), mouse anti-microtubule-associated protein 2 (MAP2; 1∶500; Chemicon; Schwalbach, Germany), goat anti-myelin associated protein (MAG; 1∶100; R & D Systems), rat anti-myelin basic protein (MBP; 1∶100; Chemicon), mouse anti-nestin (1∶50; DSHB, USA), mouse anti-neuron-specific nuclear antigen (NeuN; 1∶100; Chemicon), rabbit anti-*Pax6* (1∶300; Covance, Munich, Germany), rabbit anti-recoverin (1∶5000; Millipore, Schwalbach, Germany), mouse anti-rhodopsin Ret-P1 (1∶10000; Sigma-Aldrich), rabbit anti-Sox2 (1∶1000; Chemicon), rabbit anti-β-III-tubulin (1∶4000; Covance), and secondary Cy2-, Cy3- or Cy5-conjugated goat anti-mouse, anti-rabbit, donkey anti-goat or goat anti-rat antibodies (1∶1000 each; Jackson IR, Suffolk, UK).

### Microscopy for Immunofluorescence Examination and Cell Counting

Cells fixed on coverslips, retinal sections and flat mounted retinas were examined following immunostaining with a Z1-Imager fluorescence microscope with ApoTome (Zeiss, Jena, Germany) or a laser scanning microscope (LSM 510 META, Zeiss).

For quantification three coverslips of three independent experiments per condition were analyzed following immunostaining. For each coverslip cells from three separate, randomly chosen microscopic fields with a defined area were counted and the data subjected to statistical analysis. Results are presented as mean values ± SEM (standard error of the mean) and significance was calculated by unpaired, 2-tailed, student’s T-test using AxioVision (Zeiss), Microsoft Office Excel (Microsoft), or iWorks (Apple) software.

### Electron and Light Microscopy

Samples were perfusion-fixed in 0.1 M phosphate buffer (pH 7.4) containing 2.5% glutaraldehyde and 4% paraformaldehyde, and postfixed in 1% osmium tetroxide in water for 1 h. Samples were dehydrated through a graded series of ethanol and embedded in Epon. Semithin sections (200 nm) were stained with toluidine blue. Ultrathin sections (70 nm) were stained with 2% uranyl acetate followed by lead citrate and imaged with a TECNAI 12 transmission electron microscope (FEI) operated at 100 kV [90].
